# Establishing a molecular toolbox of lineage-specific real-time RT-PCR assays for the characterization of foot-and-mouth disease viruses circulating in Asia

**DOI:** 10.3389/fvets.2023.1271690

**Published:** 2023-11-30

**Authors:** Meruyert A. Saduakassova, Britta A. Wood, Elisabeth Henry, Ashley R. Gray, Valérie Mioulet, Akhmetzhan A. Sultanov, Jemma Wadsworth, Nick J. Knowles, Antonello Di Nardo, Donald P. King, Katarzyna Bachanek-Bankowska

**Affiliations:** ^1^FAO World Reference Laboratory for Foot-and-Mouth Disease, The Pirbright Institute, Woking, United Kingdom; ^2^Virology Department, Kazakh Scientific Research Veterinary Institute, Almaty, Kazakhstan

**Keywords:** foot-and-mouth disease virus, real-time RT-PCR, lineage-specific assays, Asia, molecular toolbox

## Abstract

Foot-and-mouth disease (FMD) is endemic in many Asian countries, with outbreaks occurring regularly due to viruses from serotypes O, A, and Asia1 that co-circulate in the region. The ability to rapidly characterize new virus occurrences provides critical information to understand the epidemiology and risks associated with field outbreaks, and helps in the selection of appropriate vaccines to control the disease. FMD lineage-specific characterization is usually determined through sequencing; however, this capacity is not always readily available. In this study, we provide a panel of real-time RT-PCR (rRT-PCR) assays to allow differentiation of the FMD virus (FMDV) lineages known to have been co-circulating in Asia during 2020. This panel included five new rRT-PCR assays designed to detect lineages O/ME-SA/PanAsia-PanAsia-2, O/ME-SA/Ind-2001, O/SEA/Mya-98, O/CATHAY, and A/ASIA/Sea-97, along with three published rRT-PCR assays for A/ASIA/Iran-05, A/ASIA/G-VII, and Asia1 serotypes. Samples of known FMD lineage (*n* = 85) were tested in parallel with all eight lineage-specific assays and an established 3D pan-FMD rRT-PCR assay, and comparative limit of detection (LOD) experiments were conducted for the five newly developed assays. All samples (85/85) were assigned to the correct serotype, and the correct lineage was assigned for 70 out of 85 samples where amplification only occurred with the homologous assay. For 13 out of 85 of the samples, there was amplification in two assays; however, the correct lineage could be designated based on the strongest Ct values for 12 out of 13 samples. An incorrect lineage was assigned for 3 out of 85 samples. The amplification efficiencies for the five new rRT-PCR assays ranged between 79.7 and 100.5%, with nucleic acid dilution experiments demonstrating broadly equivalent limits of detection when compared to the 3D pan-FMD rRT-PCR assay. These new tests, together with other published lineage-specific rRT-PCR assays, constitute a panel of assays (or molecular toolbox) that can be selected for use in FMD endemic countries (individually or a subset of the assays depending on region/lineages known to be circulating) for rapid characterization of the FMDV lineages circulating in Asia at a relatively low cost. This molecular toolbox will enhance the ability of national laboratories in endemic settings to accurately characterize circulating FMDV strains and facilitate prompt implementation of control strategies, and may be particularly useful in settings where it is difficult to access sequencing capability.

## 1 Introduction

Foot-and-mouth-disease (FMD) is a highly contagious viral disease of cloven-footed livestock and wildlife. Even though the mortality rate is typically low in adult animals, FMD has significant impacts on farmers and national economies, including decreased milk production, costs of diagnostic testing, costs of vaccination and control, and restrictions on the trade of animals and animal products (1). The causative agent, FMD virus (FMDV; family Picornaviridae, genus *Aphthovirus*), is classified into seven antigenically different serotypes [O, A, C, Asia1, Southern African Territories (SAT) 1, SAT2, and SAT3] (2), with multiple variants occurring within each serotype (3). The non-uniform geographical distribution of these variants has been classified into seven pools based on ecology, livestock farming systems, and trade, along with cultural similarities (4). Nonetheless, long-distance trans-pool movement of FMD viruses has been documented [e.g., O/ME-SA/Ind-2001 (5), A/ASIA/G-VII (6), and A/AFRICA/G-IV (7)], posing additional challenges for the control of FMD.

There are three FMD endemic pools in Asia: Pool 1, which spans east and mainland southeast Asia; Pool 2, which is confined within southern Asia (India, Nepal, and Bangladesh); and Pool 3, which spans western Asia, including regions of the Middle East (4). FMD viruses that circulate within these pools have been classified into lineages/sublineages based on the phylogenetic relationship of the viral protein 1 (VP1) coding region of the virus capsid (3, 8). Specific lineages within serotypes O, A, and Asia1 include O/ME-SA/PanAsia, O/ME-SA/PanAsia-2, O/ME-SA/Ind-2001, O/SEA/Mya-98, O/CATHAY, A/ASIA/Iran-05, A/ASIA/G-VII, A/ASIA/Sea-97, and Asia1/ASIA/Sindh-08 (6, 9, 10).

FMDV characterization is usually determined using viral genome sequencing (11, 12) and subsequent data analysis. In FMD endemic regions, sequencing capability is not always readily available, and the lack of these data can constrain effective response and control of outbreaks. Lineage-specific real-time RT-PCR (rRT-PCR) assays are an alternative method for rapid characterization of FMDV lineages, which, compared to sequencing, is both lower in cost and more likely to be accessible in endemic countries using already existing equipment. Previously, such assays have been developed to detect FMDV lineages circulating within geographical regions [Middle East (13), West Eurasia (14), and East Africa (15)] or to detect particular lineages of interest [A/ASIA/G-VII (16), SAT2/VII (17), and O/ME-SA/Ind-2001 (5)]. This study presents the development and evaluation of five new lineage-specific rRT-PCR assays that, when combined with three published assays (13, 14, 16), encompass the FMDV lineages circulating in Asia during 2020.

## 2 Materials and methods

Experiments were conducted in high-containment laboratories at The Pirbright Institute, United Kingdom, that meet the *Minimum Biorisk Management Standards for Laboratories Working with Foot-and-Mouth Disease Virus* of the European Commission for the Control of Foot-and-Mouth Disease (18).

### 2.1 Primers and probes

The molecular toolbox includes eight rRT-PCR assays designed to target the variable VP1 coding regions of specific FMDV serotypes/lineages. Five new primer and probe sets were designed to detect the following FMDV lineages: O/ME-SA/PanAsia-PanAsia-2, O/ME-SA/Ind-2001, O/SEA/Mya-98, O/CATHAY, and A/ASIA/Sea-97 (Table 1). Three other assays included in the molecular toolbox were A/ASIA/Iran-05 (14), A/ASIA/G-VII (16), and serotype Asia1 (subgroups 1, 2, and 6) (13). The newly designed primers and probes were selected by aligning VP1 coding sequences obtained from GenBank (using BioEdit Software), and then identifying a unique linear-specific target region with high intra-lineage sequence identity. Samples were also tested in parallel using an established pan-FMD assay that targets the highly conserved 3D region of the FMDV genome (19). Oligonucleotides were synthesized by either Sigma-Aldrich, Merck (St. Louis, MO, USA) or Applied Biosystems, Thermo Fisher Scientific (Waltham, MA, USA); probes were labeled with FAM and then either TAMRA or black hole quencher-1 (BHQ1) (5′ and 3′, respectively; Table 1).

**Table 1:** Primers and probes for FMDV lineages circulating in Asia.

**Serotype**	**Lineage**	**Oligo name**	**Sequence (5^′^-3^′^)**	**References**
O	ME-SA/PanAsia- PanAsia-2	FWD O-PA-FP1 (sense)	TGGACCTGATGCARACCCC	this study
		REV O-PA-RP1 (antisense)	TCGTGTTTCACTGCCACYTC	
		Probe O-PA-P2 (sense)	FAM-CTCCGCACYGCCACCTAC-TAMRA	
	ME-SA/Ind-2001	FWD IND-2001d FPv3 (sense)	GAAGAGGGCCGARACATAC	this study
		REV IND-2001d RPv2 (antisense)	GCCACAATCTTYTGYTTGTG	
		Probe IND-2001d Pv2 (sense)	FAM-CTGCTSGCCATTCACCCG-TAMRA	
	SEA/Mya-98	FWD O-Mya98-FP2 (sense)	CTGGGTGCCAAATGGAGCA	this study
		REV O-Mya98-RP3 (antisense)	CACGGTGTGGTGMCGTGT	
		Probe O-Mya98-P6 (sense)	FAM-ACCACCAACCCAACGGCRTAC-TAMRA	
	CATHAY	FWD O-CathFP3 (sense)	GATGCARATCCCTGCYCAC	this study
		REV O-CathRP3 (antisense)	ACCCARGTGAGRTCGCC	
		Probe O-CathP2 (sense)	FAM-CTGCGGACGGCCACCTACTT-TAMRA	
A	ASIA/Iran-05	FWD A-JB-F (sense)	GCCACGACCATCCACGAGCT	(14)
		REV A-JB-R (antisense)	GTCCTGYGACRACACTTCCAC	
		Probe A-JB-F-P (sense)	FAM-CTCGTGCGYATGAAACGTGCYGAGCT-TAMRA	
	ASIA/G-VII	FWD G-VII_FP (sense)	TGCTCAACTCCCTGCCTC	(16)
		REV G-VII_RP (antisense)	GAGTTCGGCACGCTTCAT	
		Probe G-VII_P (sense)	FAM-CCACYACCATCCACGAGCTG-BHQ1	
	ASIA/Sea-97	FWD A-Sea-97_ FP2 (sense)	GGACAGGTTYGTGCARATC	this study
		REV A-Sea-97_RP2 (antisense)	CACCACAATCTCAAGATCAGA	
		Probe A-Sea-97_P2 (sense)	FAM-GCGCGCGGCYACCTACTACTTT-TAMRA	
Asia1	–	FWD Asia1forward3 (sense)	GCAGTWAAGGCYGAGASCATYAC	(13)
		REV Asia1reverse2 (antisense)	GCARAGGCCTAGGGCAGTATG	
		Probe Asia1probe4 (sense)	FAM-AGCTGTTGATCCGCATGAAACGYGCG-TAMRA	

Probes for the five new assays were initially designed and tested with BHQ1 (data not shown); however, as these assays are intended for use in endemic countries where funds may be limited, the decision was made to switch to a cheaper option with TAMRA as the quencher. Nonetheless, A/ASIA/G-VII samples with mismatches in the probe binding site were undetected with TAMRA-labeled probes, and yet, these same samples were detected with BHQ1 probes (data not shown).

### 2.2 FMDV samples

Epithelium suspensions and virus isolates derived from FMDV clinical samples (*n* = 85) were selected from the archive held at the Food and Agriculture Organization (FAO) of the United Nations World Reference Laboratory for FMD (WRLFMD), The Pirbright Institute, United Kingdom. These viruses, which had been previously characterized by antigen ELISA and VP1 sequencing at the WRLFMD (for detection and genotyping reports see: https://www.wrlfmd.org/country-reports), were selected to represent the recent (2010–2020) genetic diversity in Asia within each serotype/lineage, along with representative viruses for serotypes not previously reported in Asia (Supplementary Table 1).

### 2.3 RNA extraction

All samples (*n* = 85) were processed in class II biological safety cabinets and added to lysis buffer in preparation for extraction, as per the manufacturer's guidelines, with any exceptions noted [5:13 ratio for sample:lysis/binding solution for the MagMAX™-96 Viral RNA Isolation kit (discontinued 2020; Applied Biosystems) or 2:5 ratio for sample:buffer VXL mixture, except without MagAttract Suspension G added at this stage, for the IndiMag^®^ Pathogen Kit (catalog # SP947457; INDICAL Bioscience GmbH, Leipzig, Germany)]. The majority of the samples were extracted using the MagMAX™-96 Viral RNA Isolation Kit as part of a separate study, and the extracted material had been stored at −80°C until its use in this study. The remaining samples were selected from the WRLFMD archive, extracted using the IndiMag^®^ Pathogen Kit and tested on the day of extraction. All nucleic acid extractions were performed on MagMAX™ Express-96/KingFisher™ Flex extraction robots (Thermo Fisher Scientific) according to the extraction kit protocols.

### 2.4 Positive RNA controls

Representative isolates (*n* = 9) were selected to be used as positive controls for the pan-FMD and lineage-specific assays (Supplementary Table 2). After extraction, a 10^−1^-10^−5^ dilution series was prepared for each sample [diluted in 1,000 carrier RNA (QIAGEN, Hilden, Germany) in nuclease-free water (Ambion, Thermo Fisher Scientific)] and tested in the respective assay to identify the dilution that generated Ct values of 22 ± 3. Single-use RNA aliquots were then prepared at the selected dilution and stored at −80°C until use.

### 2.5 Real-time RT-PCR

All rRT-PCR assays were performed using the EXPRESS One-Step SuperScript qRT-PCR Mix (Invitrogen, Thermo Fisher Scientific). In each reaction, 5 μL of RNA was added to 15 μL of master mix [1 μL of each primer (20 μM), 0.5 μL of the probe (15 μM), 0.5 μL of ROX reference dye diluted 1:10 with nuclease-free water, 10 μL of EXPRESS mix, and 2 μL of EXPRESS enzyme mix]. The rRT-PCRs were run on 7,500 Fast Real-time PCR instruments (Applied Biosystems) with the following cycling conditions: 50°C for 15 min, 95°C for 20 s, and then 50 cycles of 95°C for 3 s and 60°C for 30 s. Fluorescence was measured at the end of the 60°C annealing/extension step.

### 2.6 Performance of the tests using FMDV-positive samples

In the study, 85 FMDV-positive samples previously characterized as lineages O/ME-SA/PanAsia, O/ME-SA/PanAsia-2, O/ME-SA/Ind-2001, O/SEA/Mya-98, O/CATHAY, A/ASIA/Sea-97, A/ASIA/Iran-05, A/ASIA/G-VII, or serotype Asia1 were tested simultaneously in the eight lineage-specific assays and in the 3D pan-FMD assay. Additionally, eight FMDV-positive samples previously characterized as serotypes C, SAT1, SAT2, and SAT3 (two samples per serotype) were tested simultaneously in the eight lineage-specific assays and in the 3D pan-FMD assay. For each sample, the extracted RNA was diluted with nuclease-free water (70 μL RNA + 30 μL nuclease-free water; Ambion) to obtain sufficient volume to test each sample in duplicate wells in all nine assays. Each assay also included two no template control (NTC) wells and two lineage-specific/3D-positive control wells. For the test results to be accepted, all nine positive controls needed to generate Ct values, and the NTCs needed to be negative/undetected. Average Ct values are reported unless otherwise indicated. If a sample had Ct values in multiple lineage-specific assays, it was anticipated that the Ct values would be lower (i.e. stronger) in the homologous assay than in the heterologous assay [i.e., similar to previous studies that evaluated serotype-specific rRT-PCR assays with clinical samples (13, 15, 20)].

### 2.7 Comparative limit of detection

The limit of detection (LOD) for each of the five newly developed assays (O/ME-SA/PanAsia-PanAsia-2, O/ME-SA/Ind-2001, O/SEA/Mya-98, O/CATHAY, and A/ASIA/Sea-97) was assessed against the established 3D pan-FMD assay. A 10-fold dilution series was prepared for representative samples of extracted nucleic acid from each virus lineage [diluted in 1/1,000 carrier RNA (QIAGEN) in nuclease-free water (Ambion)] (Supplementary Table 2). These dilutions were tested in triplicate in both the lineage-specific and 3D pan-FMD assays in parallel. The amplification efficiency *e* for each assay was estimated using the following equation:


$$e=100\times (10^{-(1/\beta )}-1)$$

The slope β is estimated from a standard curve quantification using the linear regression parameterization of the resulting Ct values *y* vs. log of serial dilutions *x*:


$$\begin{array}{l} y=\beta x+\epsilon \end{array}$$

(https://www.thermofisher.com/uk/en/home/brands/thermo-scientific/molecular-biology/molecular-biology-learning-center/molecular-biology-resource-library/thermo-scientific-web-tools/qpcr-efficiency-calculator.html).

## 3 Results

### 3.1 Performance of the tests using FMDV-positive samples

**Figure 1:**
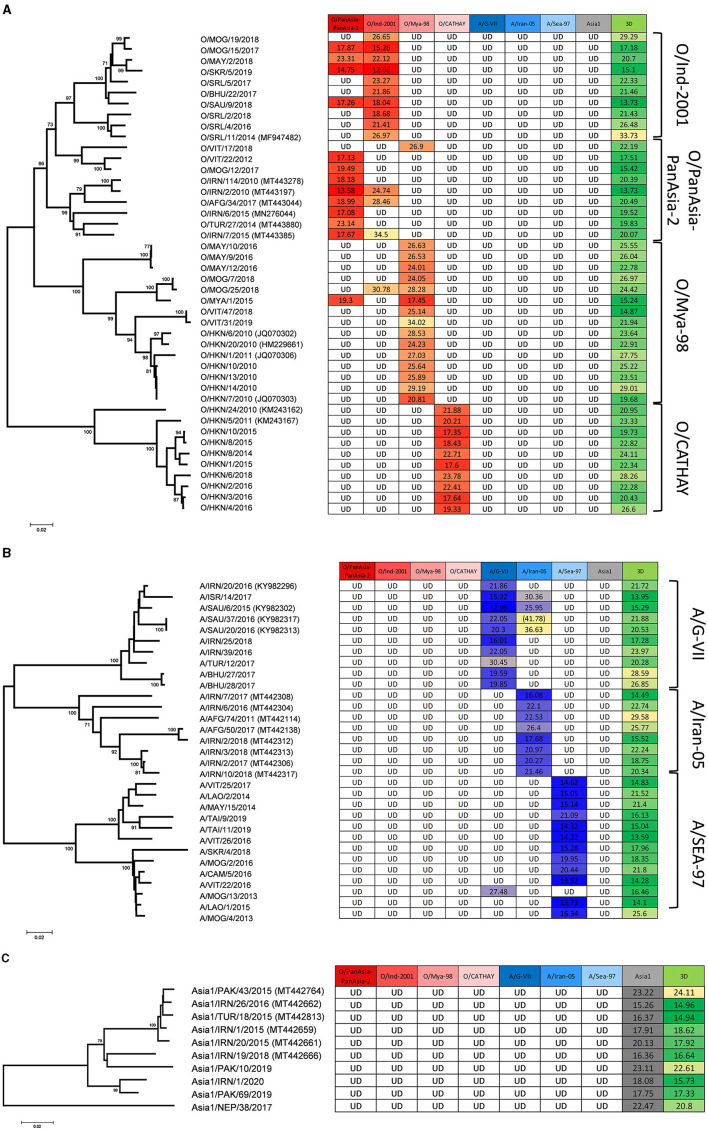
Parallel testing of FMDV samples using eight lineage-specific rRT-PCR assays. Tables display Ct values for each assay for **(A)** serotype O (red), **(B)** serotype A (blue), and **(C)** serotype Asia 1 (gray), along with the 3D pan-FMD assay (green). For each serotype, the tree shows the genetic relationship (with bootstrap values) among VP1 sequences for the samples tested within each lineage based on neighbor-joining analyses. Average Ct values are reported, except for values in parentheses where only one well-generated the Ct value shown. UD, Undetermined denotes that there was no amplification/Ct values obtained.

Of the FMDV-positive samples tested to represent the eight Asia lineages circulating between 2010 and 2020 (at least eight per lineage), all (85/85) were assigned the correct serotype with the lineage-specific rRT-PCR assays as previously categorized by antigen ELISA and VP1 sequencing (Figures 1A–C; Table 2), and the correct lineage was assigned for 70 out of 85 samples where there was amplification only in the homologous assay. For 13 out of 85 samples, there was amplification in two assays; however, the correct lineage could be designated on Ct values for 12 out of 13 samples. For 3 out of 85 samples (O/VIT/17/2018, O/SAU/9/2018, and A/MOG/13/2013; Figures 1A, B), an incorrect lineage was identified. O/VIT/17/2018, previously characterized by VP1 sequencing as O/ME-SA/PanAsia lineage, was detected exclusively by the O/SEA/Mya-98 lineage-specific rRT-PCR assay, and A/MOG/13/2013, previously characterized by VP1 sequencing as A/ASIA/Sea-97 lineage, was detected exclusively by the A/ASIA/G-VII assay; these results were repeated following the re-extraction and subsequent testing of these samples. For the 13 samples with Ct values in the homologous assay and a heterologous assay (Figures 1A, B), the Ct values for the homologous assay, as determined by VP1 sequencing, were lower (i.e., stronger) than the heterologous assay (ranging from 1.19 to 19.73 Ct lower), with the exception of O/SAU/9/2018. The samples tested to represent non-Asian serotypes (C, SAT1, SAT2, and SAT3) were not detected with the Asia lineage-specific assays (Supplementary Table 1).

**Table 2:** Summary of the performance of the Asia lineage-specific rRT-PCR assays.

		**Avg Ct per rRT-PCR assay (# samples)**								
		**O/PanAsia- PanAsia-2**	**O/Ind-2001**	**O/Mya-98**	**O/CATHAY**	**A/G-VII**	**A/Iran-05**	**A/Sea-97**	**Asia1**	**3D**
Sample serotype/lineage	O/PanAsia- PanAsia-2	18.16 (8)	29.23 (3)	26.90^*^ (1)						18.80 (9)
	O/Ind-2001	18.30 (4)	20.69 (10)							22.14 (10)
	O/Mya-98	19.30 (1)	30.78 (1)	25.83 (15)						23.30 (15)
	O/CATHAY				20.13 (10)					23.09 (10)
	A/G-VII					20.04 (10)	34.71 (4)			21.03 (10)
	A/Iran-05						20.94 (8)			21.18 (8)
	A/Sea-97					27.48^*^ (1)		16.17 (12)		17.77 (13)
	Asia1								19.07 (10)	18.36 (10)

Sample lineage was determined by VP1 sequencing; all samples were detected with the 3D pan-FMD assay (*n* = 85). Average Ct values and the number of samples detected in the respective homologous assays are highlighted in black; samples that also had amplification in non-homologous assays are highlighted in gray. Two samples (^*^) were only detected with a non-homologous assay.

### 3.2 Comparative limit of detection

**Figure 2:**
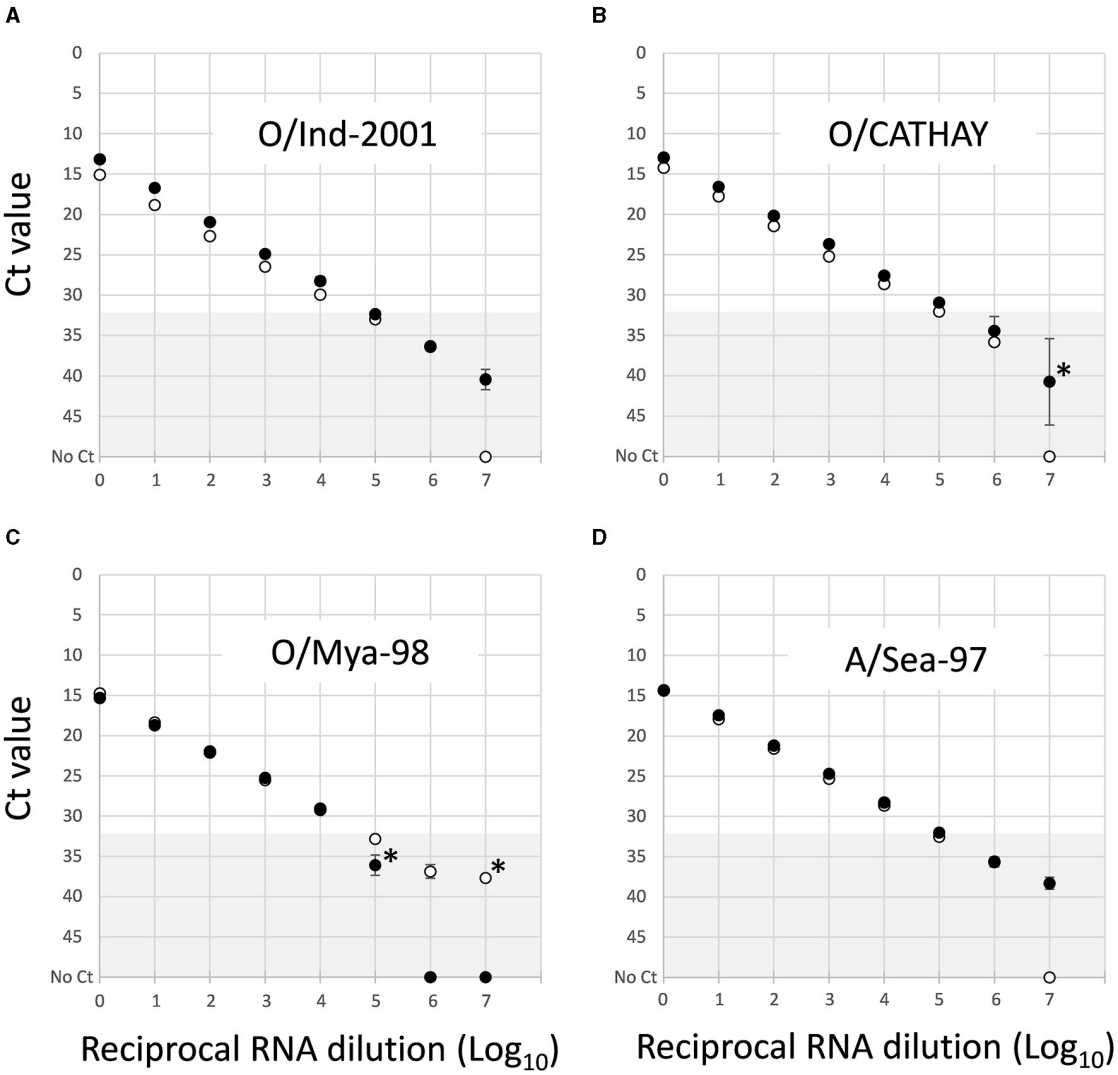
Comparative limit of detection for rRT-PCR assays that detect **(A)** O/ME-SA/Ind-2001, **(B)** O/CATHAY, **(C)** O/SEA/Mya-98, and **(D)** A/ASIA/Sea-97 using representative FMDV isolates. Data shown represent mean Ct values ± SD (*n* = 3) for parallel testing of Log_10_ nucleic acid dilutions by the lineage-specific (•) and reference 3D pan-FMD assays (◦). Dilutions where individual replicates failed to amplify are shown *. The gray shading (Ct 32–50) denotes values above the 32 Ct cut off used to define positive samples for the 3D pan-FMD assay.

The LOD (representing the highest dilution at which FMD viral RNA could be detected) for each of the five newly developed assays were assessed compared to that of the 3D pan-FMD assay, which has demonstrated 99.9–100% diagnostic specificity for negative samples at the Ct cutoff value of 32 (21). The decimal dilutions of the nucleic acid prepared for representative isolates of O/ME-SA/Ind2001, O/CATHAY, O/SEA/Mya-98, and A/ASIA/Sea-97 lineages showed responses similar to the 3D pan-FMD assay (Figure 2). Furthermore, for these four assays, the highest dilution where FMD viral RNA could be detected was approximately the same when a Ct cutoff value of 32 was applied for the samples tested. Based on these dilution series results, the amplification efficiencies were calculated to be 82.1, 89.9, 100.5, and 92.3% for the O/ME-SA/Ind2001, O/CATHAY, O/SEA/May-98, and A/ASIA/Sea-97 assays, respectively. In comparison, the amplification efficiency for the 3D pan-FMD assay for the same samples was between 89.1 and 89.9%.

**Figure 3:**
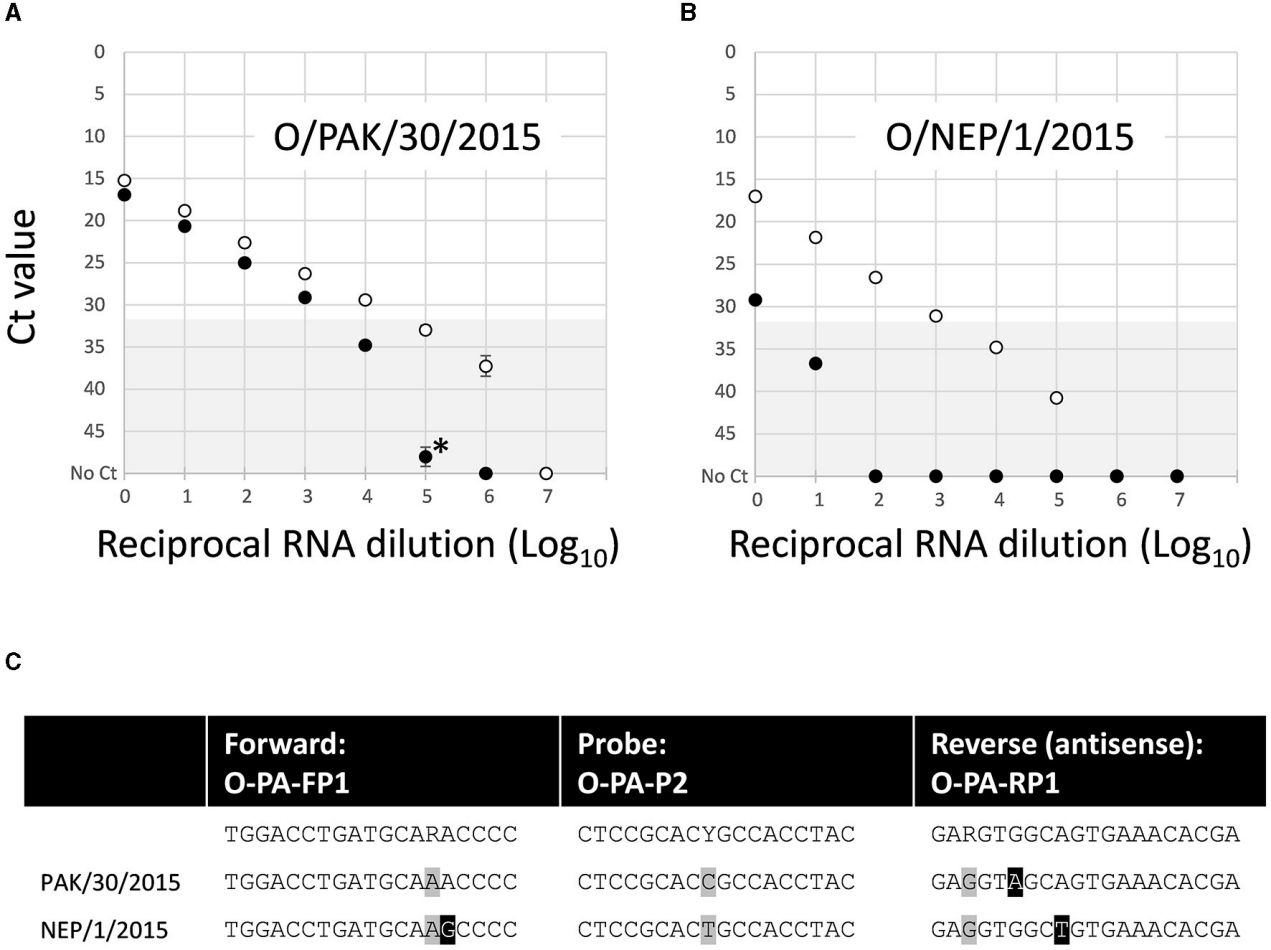
Comparative limit of detection for the O/ME-SA/PanAsia-PanAsia-2 rRT-PCR assay using nucleic acid for two representative FMDV isolates: **(A)** O/PAK/30/2015 and **(B)** O/NEP/1/2015. **(C)** Sequences for the primer and probe binding sites indicate two nucleotide substitutions, highlighted in black boxes for O/NEP/1/2015, while additional variant positions that are accommodated by degenerate sites in the assay oligonucleotide sequences are highlighted by gray boxes. The gray shading (Ct 32–50) denotes values above the 32 Ct cut off used to define positive samples for the 3D pan-FMD assay.

Similar analyses for the O/ME-SA/PanAsia-PanAsia-2 assay showed a lower LOD for the lineage-specific test compared to the 3D pan-FMD assay. One isolate (O/PAK/30/2015), which has a single nucleotide substitution in the reverse primer binding site, demonstrated ~10-fold reduced LOD compared to the 3D pan-FMD assay (Figure 3A). In contrast, a second isolate (O/NEP/1/2015), which has two nucleotide substitutions in primer binding sites (one forward and one reverse), demonstrated a markedly reduced LOD in the lineage-specific test compared to the 3D pan-FMD assay (Figure 3B). For the O/ME-SA/PanAsia-PanAsia-2 assay, the amplification efficiency was estimated within a range of 76.7–82.3%.

## 4 Discussion

This study presents the development of five new lineage-specific rRT-PCR assays that, when combined with three published lineage-specific assays, can be used to differentiate FMDVs circulating within the endemic pools of Asia during 2020. The three Asian FMD endemic pools [Pools 1–3; (4)] maintain different FMDV lineages with unequal geographical distribution. Certain FMDV lineages are widely distributed (e.g., O/ME-SA/Ind-2001), while others have a more restricted range [e.g., O/CATHAY; (22)]. Laboratories that already have the capacity to perform pan-FMD rRT-PCR assays (defined in the OIE/WOAH manual) only need to obtain oligonucleotides for the lineage-specific assays presented here, as all other master mix components and thermocycling conditions remain unchanged. This generic format enables laboratories to select assays that are most suitable to cover the different virus lineages and threats in their country/region (Supplementary Figure 1) and, where appropriate, allows further inclusion of other rRT-PCR assays (e.g., SAT2/XIV lineage-specific assay developed in 2023 in response to outbreaks in the Middle East; see https://www.foot-and-mouth.org/science/lineage-specific-pcr). Further development may be warranted to move toward multiplex formats and/or validations of assay-specific cutoffs. Nonetheless, these assays already have the potential to quickly provide serotypic and/or lineage-specific data during FMD outbreaks. Where possible, representative samples from FMDV outbreaks should be sequenced to trace the origin of these lineages phylogenetically and for samples that are FMDV genome positive (e.g., 3D pan-FMD assay) but unable to characterize the virus with the lineage-specific rRT-PCR assays.

This study analyzed 85 FMDV-positive samples representing the lineages of serotypes O, A, and Asia1 circulating in Asia from 2010 to 2020. When tested in parallel, the assays correctly defined all serotypes, while the specific FMDV lineage was correctly assigned for 82 (96.5%) of these samples. There were 13 occasions (15.3% of samples) where a sample generated a signal in more than one lineage-specific assay. For these samples, the Ct values of the homologous assays were typically lower (ranging from 1.19 to 19.73), with the exception of O/SAU/9/2018; for O/SAU/9/2018 the average difference in Ct was 0.78 and likely within the expected error. Additionally, the homologous assay typically generated a sigmoidal curve, and on the multicomponent plot had a higher increase in fluorescence compared to the heterologous assay. Nonetheless, results such as these complicate lineage assignment based on rRT-PCR alone, potentially leading to incorrect lineage assignments, especially if not all lineage-specific assays within a serotype are tested. In this context, it is possible for a host to have a mixed infection in epidemiological systems where multiple (and different) FMDV lineages are co-circulating, thus generating multiple signals on rRT-PCR assays. In such cases, VP1 sequencing is crucial in determining whether the results obtained from lineage-specific assays are accurate or if there is non-specific amplification.

The viruses included in these experiments demonstrate the use of the lineage-specific assays and do not characterize the full extent of the genetic diversity of the FMDV lineages circulating in Asia. The challenge of designing lineage-specific tests was most apparent for the O/ME-SA/PanAsia and O/ME-SA/PanAsia-2 lineages where the high degree of sequence identity [min-max range 68.72–97.00%, median 91.78% (PI 88.47–94.47%)] precluded the design of individual tests. Furthermore, three O/ME-SA/PanAsia-2 samples cross-reacted with the O/ME-SA/Ind-2001 assay, and four O/ME-SA/Ind-2001 (specially, Ind-2001e lineage) samples cross-reacted with the O/ME-SA/PanAsia-PanAsia-2 assay, one of which had the lowest Ct value for the heterologous assay (O/SAU/9/2018; Figure 1A; Supplementary Table 1). These findings highlight the need to include multiple lineage-specific assays when determining the virus lineage based on rRT-PCR results alone. Furthermore, these data reinforce the importance of continuously monitoring the performance of these assays to ensure that primers and probes are suitable for the intended purpose. These assays are designed to target the VP1 coding sequence, which is the region of the genome that genetically types the serotype and lineage of an FMDV-positive sample. As such, constant evolutionary changes in VP1 might inevitably affect the performance of these assays due to increasing genomic variability over time. Therefore, the design of the rRT-PCR primers and probes must balance the requirements for high intra-strain sensitivity and low inter-strain cross-reactivity.

Dilution series were used for assessing the LOD for the new assays in comparison to the established rRT-PCR that targets the highly conserved 3D region of the FMDV genome. This approach, although not providing absolute analytical sensitivities for these tests, enables a direct comparison between the reference (3D pan-FMD) and each lineage-specific assay by using a common template dilution series. This minimizes errors during the preparation of separate tailored standards. For some of the samples tested, there was a close agreement between LOD estimates for the lineage-specific and 3D pan-FMD assays (O/ME-SA/Ind-2001, O/CATHAY, O/SEA/Mya-98, and A/ASIA/Sea-97; Figure 2). However, when additional samples were tested for the O/ME-SA/PanAsia-PanAsia-2, O/SA/Ind-2001, and O/CATHAY assays, there was variability in the LODs, even when the virus sequences were identical with each other and/or had no mismatches compared to the primers and probes used in these tests (data not shown). The specific reasons for these differences are not fully understood but could be attributed to sequence heterogeneity among these viruses (including variations within the degenerate sites for the primers and probes) or subtle differences in the RNA folding of their genomes. These data presented for O/NEP/1/2015 (Figure 3B) represent the greatest differences between the LOD estimated for a lineage-specific test and the 3D pan-FMD rRT-PCR across all of the samples tested.

Unexpected sequence mismatches have been shown to negatively impact the performance of another pan-FMD rRT-PCR that targets the 5′UTR of FMDV (23), and *in silico* tools have been designed to estimate the impacts of these mismatches (24). The results presented here emphasize the importance of continuously sequencing representative viruses to ensure that the assays are up to date and suitable for the intended purpose of detecting circulating strains, and where needed, primers and/or probes can be modified with the evolution of field strains. It is advisable to conduct a well-validated pan-FMD assay, such as the Callahan 3D assay, in parallel with lineage-specific assays to ensure that FMDV-positive samples are not falsely designated as negative if not detected in a lineage-specific assay.

The five newly introduced lineage-specific assays, along with the three already published assays, constitute a comprehensive panel of assays (or molecular toolbox) for the rapid characterization of FMDV lineages circulating in Asia at a relatively low cost. This molecular toolbox will enhance the ability of national laboratories in endemic settings to accurately characterize currently circulating FMDV strains, and thus facilitating the prompt implementation of control strategies.

## Data availability statement

The raw data supporting the conclusions of this article will be made available by the authors, without undue reservation.

## Ethics statement

Ethical approval was not required for the study involving animals in accordance with the local legislation and institutional requirements because samples described in the paper were collected as part of routine veterinary investigations and submitted to national/international reference laboratories for FMDV testing.

## Author contributions

MS: Methodology, Writing—review & editing, Investigation, Writing—original draft. BW: Investigation, Methodology, Visualization, Writing—original draft, Writing—review & editing, Conceptualization, Formal analysis. EH: Investigation, Writing—review & editing. AG: Investigation, Writing—review & editing. VM: Methodology, Validation, Writing—review & editing, Conceptualization. AS: Funding acquisition, Writing—review & editing, Conceptualization. JW: Investigation, Writing—review & editing. NK: Visualization, Writing—review & editing, Formal analysis. AD: Visualization, Writing—review & editing, Formal analysis. DK: Funding acquisition, Visualization, Writing—original draft, Writing—review & editing, Conceptualization, Formal analysis. KB-B: Conceptualization, Writing—review & editing, Methodology, Writing—original draft, Funding acquisition, Investigation.

## Supplementary material

Supplementary Figure 1 Conjectured distribution of FMDV lineages in Asia to guide the selection of appropriate assays. Maps collate data from the WOAH/FAO FMD Laboratory Network to show where the following FMDV lineage might be present: **(A)** O/ME-SA/Ind-2001, **(B)** O/ME-SA/PanAsia-2 (west) and O/ME-SA/PanAsia (east), **(C)** O/SEA/May-98, **(D)** O/CATHAY, **(E)** A/ASIA/Sea-97, **(F)** A/ASIA/Iran-05, **(G)** A/ASIA/G-VII, and **(H)** serotype Asia 1.

Supplementary Table 1 Sample details, including topotype/lineage, species, sample matrix, and GenBank accession numbers. Sample matrices include original epithelium suspension (OS) or virus isolates. Isolates were propagated in various cell lines, including primary bovine thyroid cells (BYT), a fetal porcine kidney cell line (LFBK), a bovine vial diarrhea virus-free porcine kidney cell line (WRL-LFBK), a porcine kidney cell line (IB-RS2, abbreviated as RS), and baby hamster kidney fibroblasts (BHK) [R and B cells are unknown/as reported by the sender], followed by the number of passages within those cells.

Supplementary Table 2 Details for the isolates used as positive controls and in the LOD experiments, including topotype/lineage, species, sample matrix, GenBank accession numbers, and lineage-specific assays. Isolates were propagated in primary bovine thyroid cells (BYT) or a fetal porcine kidney cell line (LFBK) [R and B cells are unknown/as reported by the sender], followed by the number of passages within those cells.
